# Microbial activities in soil cultivated with corn and amended with sewage sludge

**DOI:** 10.1186/s40064-016-3502-9

**Published:** 2016-10-21

**Authors:** Rosana Faria Vieira, Ricardo Antônio Almeida Pazianotto

**Affiliations:** 1 Department of Organic Matter, Embrapa Meio Ambiente, CP 69, Jaguariúna, São Paulo CEP 13820-000 Brazil; 2 Aplicated Mathematics, Embrapa Meio Ambiente, CP 69, Jaguariúna, São Paulo CEP 13820-000 Brazil

**Keywords:** Sewage sludge, Microbial activity, Metabolic quotient, Corn

## Abstract

**Background:**

One of the main concerns related to the increasing use of sewage sludge in the soil is the possible presence of excess nutrients, which could cause environmental problems and detrimental effects on the soil microorganisms, considered essential to soil nutrient cycling. Thus, the objective of this work was to evaluate the microbial biomass and activity and some chemical characteristics of one specific tropical soil, classified as Dark Red Distroferric Latosol, of a loamy/clayey texture, in a long-term field experiment using anaerobically digested household sludge amendment. The sludge doses applied were the recommended dose and 2, 4 and 8 times the recommended dose. The authors hypothesized that the frequent application of this compound to the soil, even when using the recommended dose, could affect the available phosphorus (P_av_) and heavy metal contents of the soil, resulting in concentrations above the needs of the culture as well as negatively affecting the activity of the soil microorganisms.

**Results:**

The results demonstrated that successive applications of sludge, calculated considering the recommended dose of N for corn, did not increase the soil P_av_ contents in relation to the treatment in which the fertilizer was applied considering the nutrient needs of the culture, contrary to what happened with the highest sludge doses. The Cr, Ni and Cu contents increased with increase in sludge dose, but did not surpass the limits considered inadequate. There were no accentuated differences between the treatments with respect to microbial biomass C. Basal respiration and the FDA hydrolysis were considered to be the parameters that most differentiated the effect of increasing sludge doses on the microbial activity.

**Conclusion:**

The application of a sludge dose to a tropical soil, based on the recommended dose, did not affect the P_av_ or heavy metal contents of the soil even after years of application. Since there were no differences between the treatments with respect to the C_mic_ values, to the contrary of what happened with the other microbiological parameters evaluated, the possibility of changes in the composition of the microbial community with the higher sludge doses was considered.

## Background

With the increasing need to develop sustainable agricultural practices, the use of waste products has been the target of many studies in various countries. Sewage sludge (SS), for example, is a residue rich in organic matter, generated during the treatment of residual waters in Sewage Treatment Plants (STP). Population growth allied to an expansion in industrial activity has resulted in a considerable increase in the production of this residue. Parallel to the great production of this sludge, there has been an increase in the concern to use it in a sustainable and economically viable way without damage to the environment. As a result of various inconveniences in dumping sewage sludge in landfills or incinerating it, its application in agriculture has emerged as a promising technique. The incorporation of this residue into the soil allows for better use of the nutrients by the plants, since they are in the organic form and are liberated gradually, thus providing the nutritional requirements of the plants in the most adequate way throughout the cycle of the culture (Claassen and Carey 2007). Other benefits associated with the use of sewage sludge in agricultural areas are improvements in the physical properties of the soil (Claassen and Carey 2007). Such residue acts as a cementing agent for aggregate formation and stabilization (Sundermeier et al. 2011).

The use of sludge as a nutrient source could reduce problems related to the intense application of fertilizers, such as nitrogen and phosphate fertilizers. These fertilizers provide nutrients which are quickly made available to the plants, increasing productivity of the cultures. However, they do not improve the soil health and can, in long-term, damage the soil (Hernández et al. 2016). The soil nitrogen fertilizers application has been associated with the emission of nitrous oxide and nitrate leaching connected to the greenhouse effect and to contamination of the water table, respectively (Di and Cameron 2002). The use of sludge as the nitrogen (N) source presents economic benefits, since it transforms a reject into an important agricultural consumable, reducing the manufacture of nitrogen fertilizers, which is a costly process. The use of sewage sludge as a source of phosphorus (P) is of extreme importance in agriculture, considering that its reserves are being depleted (Van Vuuren et al. 2010). However, an excessive accumulation of P in the soil in relation to that removed by the cultures could contribute to increasing the potential to lose this element to the environment increasing the risk of run off/erosion losses to surface water, resulting in eutrophication (Elliott and O’Connor 2007). It is thus important to take the P contents in the sludge into consideration when establishing the doses of residue to be applied to the soil, as already occurs in some places (Lu et al. 2012).

According to Brandt et al. (2004) excessive P loading in the soil due to the application of biosolids to the land, is one concern regarding land-based recycling programs. Ma et al. (2014) observed that the application of sewage sludge increased the phosphorus content in the 0–20 cm surface soil layer by 100–200 %. Cogger et al. (2013) observed an excessive P content in the soil even nine years after the last sludge application. According to Parat et al. (2005) the long-term application of sludge in addition to the increase in the organic C content of up to 2.5 times, also increases the phosphorus contents of the soil. As stated by Corrêa (2004), different biosolids present different capacities to supply N and P to plants and the efficiency of the biosolids as P source depends also on soil type. However, an increase in the available P (P_av_) contents due to sludge application does not always occur. Using a Red Eutroferric Latosol with a clayey texture Galdos (2003) did not observe an increase in the P_av_ of the soil after two years of applying sewage sludge, as compared to the fertilized control, even when twice the concentration recommended for corn was used. These facts suggest the need to carry out further studies on the capacity of sewage sludge to increase the P_av_ contents in different types of soil. This type of study is very important in tropical soils that are highly weathered and have high adsorption capacity of this element. Examples of these soils are the latosols, which cover immense areas in tropical regions and represent about 60 % of the important agricultural areas in Brazil (Soares and Alleoni 2008). According to Mtshali et al. (2014) because of the differences in sludge characteristics among sludges that undergo different levels of treatment as well as the extensive and variable nature of pollutant inputs to wastewater, the fertilizer potential and pollutant risk of sewage sludge intended for agricultural application has to be specifically evaluated for each sludge.

Sludge also acts as a source of heavy metals such as chromium (Cr), nickel (Ni), lead (Pb), copper (Cu) and zinc (Zn), whose contents vary according to the origin of the residue. A large fraction of them remain in the soil for many years once applied and when in excess, may be toxic to microorganisms and reduce the uptake of essential nutrients in plants (Bramryd 2013). Thus it is important to evaluate the contents of these elements in the soil more frequently in areas where the soils are supplemented with sewage sludge.

Studies on the use of sewage sludge in agriculture have been focused mainly on evaluating its role in the introduction of heavy metals in the food chain. Information on the effect of this residue on microbial activity with respect to maintaining soil quality are scares (Usman et al. 2012). To the authors knowledge there is a dearth of studies related to the effect of the application of sewage sludge on the activity of soil microorganisms in tropical regions of Brazil. The functions of microorganisms in the soil are extremely important since they regulate ecosystem process such as nutrient cycling (Mgang et al. 2016). For the phosphorus, for example, soil microorganisms act as sink and source of phosphorus (P) and mediate key processes in the cycling of this element. In the case of sludge, the results concerning the microbiological parameters can depend on other specific characteristics in addition to the nutrients contents, such as the time of exposure to the soil (MacDonald et al. 2007), the application frequency and the decomposition rate (Tam and Wong 1990), as well as the presence of organic substances that could be harmful to the soil microbial processes (Hseu 2006).

Individual parameters such as microbial biomass C (C_mic_) and N (N_mic_), basic respiration (BR) or even enzyme activities, have been widely used to measure the effects of different types of soil management on the soil microbiota (Schloter et al. 2003; Debosz et al. 2002), including areas where sludge was applied (Armenta et al. 2012; Revoredo and Melo 2007). Decreases in the microbial biomass and enzyme activities were observed in some studies due to the application of sewage sludge to the soil (Knight et al. 1997; Kao et al. 2006), whereas in other soils, amendment with sewage sludge resulted in increased soil microbial activity, soil respiration and enzyme activities (Sastre et al. 1996; Banerjee et al. 1997). However, the increase of soil respiration might indicate that soil microorganisms divert more energy from growth into maintenance as stress increase (Yan et al. 2003). Due to this the metabolic quotient (*q*CO_2_) has been used to better interpret the results. The *q*CO_2_ evaluates the community respiration per biomass carbon unit, and is a relative measure of how efficiently the soil microbial biomass is utilizing the C resources, or, in other words, it determines the degree of substrate limitation for soil microbes (Wardle and Ghani 1995). Other rations such as the C_mic_:C_org_ and N_mic_:N_tot_ have also been widely used. They reflect the contribution of microbial biomass to the soil C_org_ and total N (N_tot_), respectively (Anderson and Domsch 1989) with the expectation that higher values for these ratios result in improved soil quality. A low C_mic_:C_org_ ratio and high *q*CO_2_ generally reflect a less efficient use of organic substrates by the microbial biomass (Anderson 2003).

Due to various factors that affect the adequacy of the use of sewage sludge in agriculture, such as the soil type and origin of the residue, the objective of this work was to study how long-term applications of increasing doses of domestic sludge anaerobically digested could be affecting the nutrient contents and microbial activity of a tropical soil with loamy/clayey texture. The authors hypothesized that continued sludge applications, even when using doses considered adequate, could increase the P and heavy metal contents of the soil, resulting in concentrations above the needs of the culture as well as negatively affecting the activity of the soil microorganisms.

## Methods

**Fig. 1:**
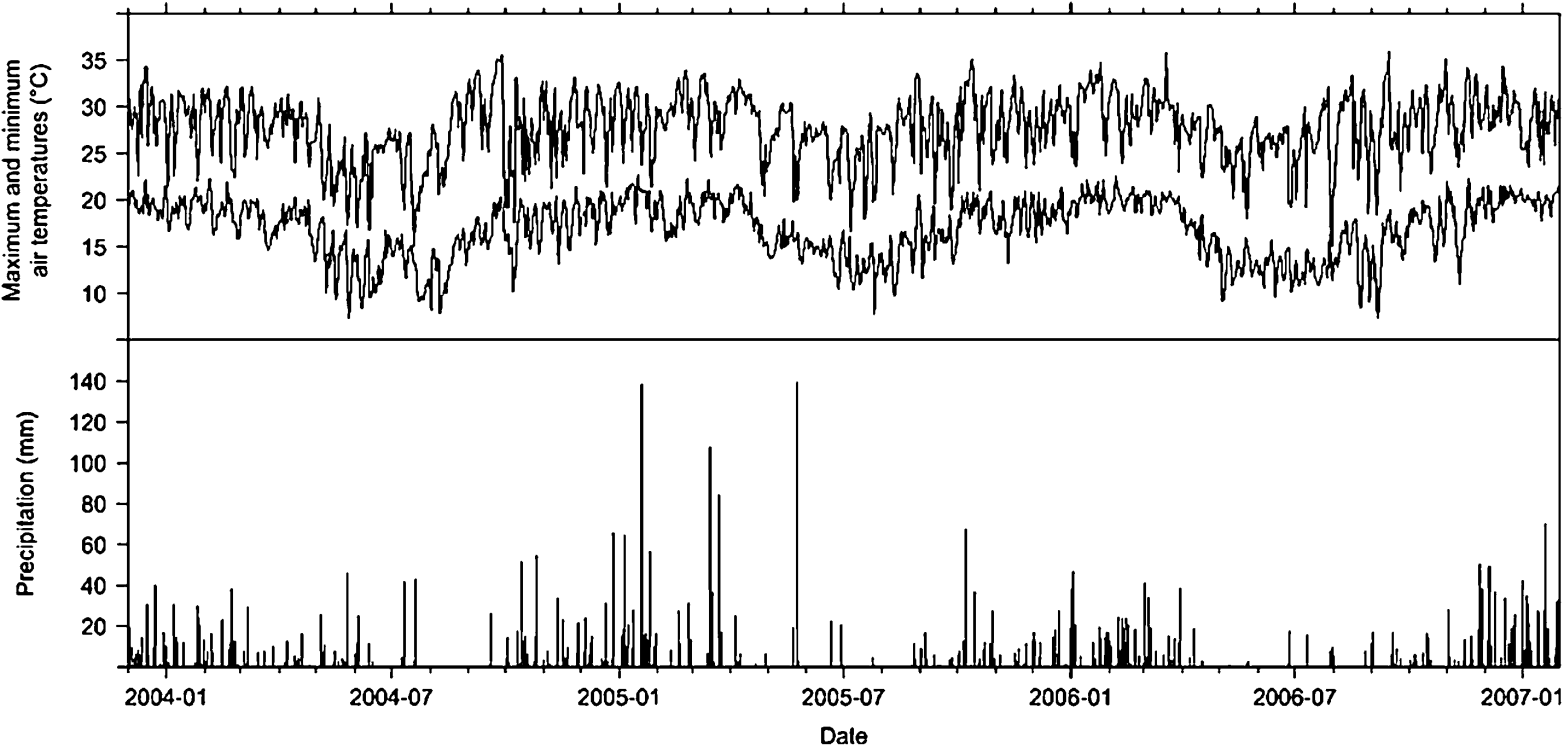
Precipitation and maximum and minimum temperature in the experiment place. 01, January; 07, July

The experiment were conducted at the Embrapa Meio Ambiente field in Jaguariúna, State of São Paulo, Brazil (latitude 22°41′S, longitude 47°W Gr. and altitude 570 m), on a Dark Red Distroferric Latossol (loamy/clayey texture). The climate is Cwa mesothermal according to the Köppen classification, which is characterized by hot summers and a dry season from May to September (Fig. 1).

The experiment was set up as a completely randomized block design with three repetitions, and included the following treatments: mineral fertilization (MF, control treatment), and doses 1 (1FS), 2 (2FS), 4 (4FS) and 8 (8FS) of sewage sludge. Dose 1 was calculated considering the recommended N application for the culture and doses 2, 4 and 8 were, respectively, two, four and eight times dose 1. The total amounts of sludge applied over the years were 24.7, 50.1, 99.1 and 198.2 t ha^−1^, respectively, for the treatments 1FS, 2FS, 4FS and 8FS. The sludge was first applied in April of 1999, and then in the month of November in 1999, 2000, 2001, 2002 and 2003. Sewage sludge was not applied in 2004 and 2005. The sewage sludge was always uniformly distributed on the soil surface and rototilled to a depth of 20 cm. This residue, that was anaerobically digested, came from the Franca sewage treatment station, in the State São Paulo, that receives household sludges. Soil management was conventional with annual plowing. The experimental plots were not irrigated and corn was the test plant used in all cultivations. After each corn harvest the soil was left fallow until the next cultivation.

Four sub-samples were randomly taken from the 0 to 20 cm soil layer of each plot in October, 2006. At this time the 2006/2007 corn crop had still not been seeded, and hence the soil had been fallow since the previous corn harvest in May. The sub-samples were thoroughly mixed together to give a single composite sample, transported to the laboratory and the analyses started within 24 h. The C_org_ content was determined using the Walkley and Black dichromate oxidation method (Nelson and Sommers 1982), and N_tot_ content by the Kjeldahl digestion procedure (Bremner 1965). The soil pH was measured in a 1.0:2.5 soil:water mixture using a glass electrode and the soil K contents were extracted with 1 M ammonium acetate (1:10 soil:extractant for 1 h) and analyzed by atomic absorption spectrophotometry (Camargo et al. 2009). Available P was evaluated using the resin method (Camargo et al. 2009). The heavy metal contents were digested using concentrated HNO_3_ and HCl according to USEPA method no. 3051a.

### Microbial biomass and activity measurements

The microbial biomass carbon and nitrogen contents were determined by the fumigation-extraction method (Vance et al. 1987; Brookes et al. 1985), using a k_EC_ factors of 0.33 and 0.54, respectively. Extractable C and N were determined in the same extract. The total C and N in the extracts were determined by the Walkley and Black and Kjeldahl methods, respectively (Nelson and Sommers 1982; Bremner 1965), maintaining the extracts frozen at −15 °C until analyzed (Hargreaves et al. 2003). For the measurement of CO_2_ evolution, 100 g of moist soil were placed at the bottom of a 1.5 l air-tight sealed jar, together with 10 ml of 0.5 N KOH. Two replicates were made for each sample and three jars with KOH but with no soil were used as the controls (Alef 1995). All the jars were incubated for 15 days at 25 ± 2 °C and the CO_2_ evolved then determined by titration. The fluorescein diacetate hydrolytic activity (FDA) was determined using the method described by Adam and Duncan (2001). The metabolic quotient (*q*CO_2_) was expressed from the ratio between basal respiration and microbial biomass C (Anderson and Domsch 1990) and the C_mic_:C_org_ and N_mic_:N_tot_ ratios expressed as the amounts of C_mic_ and N_mic_ per unit of C_org_ and N_tot_, respectively.

### Data analysis

A one-way analysis of variance (ANOVA) was carried out for each variable studied and the means separated amongst the treatments using the least significant difference (LSD) test. When the assumptions of normality and homogeneity of variance were violated, the nonparametric Friedman rank sum test with multiple comparisons of the treatments was applied and significant differences detected at the 0.05 level. The coefficients of correlation between all the variables were estimated using the Pearson method with *p* < 0.05 as the significant threshold. To reveal the similarities and differences between the samples and to assess the relationships between the variables observed, the principal component analysis (PCA) was applied to all the data. The variables of the ratios (*q*CO_2_, C_mic_:C_org_, N_mic_:N_tot_) were considered supplementary. For basal respiration, all samples for the 1FS treatment were lost and these missing values were imputed with a PCA model (Josse and Husson 2012). All analyses were detected using the R open source statistical software (R Core Team 2015).

## Results

### Soil characteristics

The results showed that both the biological and chemical properties were significantly influenced by the sludge doses applied. The C_org_ values were higher in the soils treated with the two highest sludge doses, whilst the available P (P_av_) values of the soil increased with increasing sludge doses (Table 1). The mean P_av_ contents in the treatments MF and 1FS were 3.3, 4.9 and 13.4 times smaller than those obtained in the 2FS, 4FS and 8FS treatments, respectively. This great difference obtained between the treatments with respect to the soil P content was observed even in the comparison between the treatments 1FS and 2FS, which were of 230 %. The N_tot_ showed no significant differences between the treatments MF, 1FS and 2FS and was higher in treatments 4FS and 8FS. The values for pH and K showed no consistent differences between the treatments. The contents of the soil micronutrients Cr and Cu increased with increase in sludge dose. The Zn content was lower in the 1FS treatment than in the MF treatment and varied with the sludge dose, being greatest in the 8FS treatment (Table 1). The soil Ni content was lowest in the MF treatment and showed increasing values from the 1FS to the 4FS treatments, but no significant difference was observed between the values of this element in the 4FS and 8FS treatments. The total Cd and total Pb contents of the soil, in addition to being very low, showed no significant differences between the treatments and therefore the data were not presented.

**Table 1:** Soil chemical properties collected in October, 2006

Treatments	$$ \begin{array}{*{20}l} {{\text{pH}}_{{ ( {\text{H}}_{ 2} {\text{O)}}}} } \hfill \\ \end{array} $$	P (mg kg^−1^)	K (mmol_c_kg^−1^)	C_org_ (%)	N_tot_ (%)	C/N	Cr (mg kg^−1^)	Ni (mg kg^−1^)	Zn (mg kg^−1^)	Cu (mg kg^−1^)
MF	5.83 ab	11.50 d	4.90 a	2.84 cd	0.17 b	15.57 a	21.30 e	0.60 d	4.60 c	6.77 c
1FS	6.00 a	15.30 d	3.60 ab	2.71 d	0.18 b	15.61 a	24.37 d	1.30 c	1.00 d	5.07 d
2FS	5.80 ab	50.50 c	2.40 bc	2.94 c	0.19 b	14.63 a	27.13 c	2.20 b	5.80 c	7.67 c
4FS	5.80 ab	65.80 b	2.10 c	3.15 b	0.22 ab	14.82 a	32.50 b	2.80 a	14.90 b	9.70 b
8FS	5.67 b	179.50 a	2.50 bc	3.82 a	0.26 a	17.80 a	34.07 a	3.30 a	28.30 a	11.43 a

Means followed by the same letter(s) within the same column are statistically the same at *p* ≤ 0.05 (LSD-test)

MF, mineral fertilization recommended for the crop; 1FS, sewage sludge based on the nitrogen concentration that provides the same amount of N as in the mineral fertilization; 2FS, two; 4FS, four; 8FS, eight times the N recommended sewage sludge dosage

### Microbial biomass and activity

The values found for C_mic_ were lowest for the MF treatment and highest for the 8FS treatment (Table 2), and the values found for the other sludge doses showed strong tendencies to be greater than the value obtained for the control treatment. The results obtained for N_mic_ varied from 19.33 to 29.21 μg g^−1^ of soil and were highest for the largest three sludge doses. There were no significant differences for this parameter between the MF and 1FS treatments. The biological soil activity, measured as the BR efflux, was greatest for the 4FS and 8FS treatments, showing mean values on average 52 % higher than the mean values obtained for the MF, 1FS and 2FS treatments. The FDA activities were highest for the 2FS, 4FS and 8FS treatments, and were, on average, 27 % higher than the mean values obtained for the MF and 1FS treatments (Table 2).

**Table 2:** Microbial biomass C and N, basic respiration and FDA activity as affected by the treatments

Treatments	C_mic_ (μg g^−1^ soil)	N_mic_ (μg g^−1^ soil)	BR (μg CO_2_ g^−1^ soil dia^−1^)	FDA (μg g^−1^ soil h^−1^)
MF	227 b	19.33 b	7.40 c	7.66 bc
1SF	313 ab	19.93 b	8.81 bc	7.26 c
2SF	309 ab	28.52 a	9.02 bc	8.98 ab
4SF	325 ab	29.21 a	10.57 ab	9.24 a
8SF	368 a	25.35 ab	15.05 a	10.3 a

Means followed by the same letter(s) within the same column are statistically the same at *p* ≤ 0.05 (LSD-test for C_mic_, N_mic_, FDA, multiple comparison Friedman test for BR)

MF, mineral fertilization recommended for the crop; 1FS, sewage sludge based on the nitrogen concentration that provides the same amount of N as in the mineral fertilization; 2FS, two; 4FS, four; 8FS, eight times the N recommended sewage sludge dosage. C_mic_, microbial biomass C; N_mic_, microbial biomass N; BR, basic respiration; FDA, fluorescein diacetate activity

The C_mic_:C_org_ ratio, used as a measure of the biologically labile C pool, ranged from 0.80 to 1.16 %, and the ratio obtained for the 1FS treatment was 45 % greater than that obtained for the MF treatment. Considering only the treatments with added sludge, no significant difference was found between the treatments for this parameter, although there was a tendency for it to be lowest for the treatment 8FS. The value for N_mic_ as a percentage of the total N (N_mic_:N_tot_) ranged from 0.97 to 1.47 %, with little significant difference between the treatments (Table 3). The value for *q*CO_2_ showed a strong tendency to be higher for the two largest sludge doses and for the MF treatment (Table 3). The *q*CO_2_ value obtained for treatment 8FS was 35 % higher than the mean value obtained for MF, 1FS and 2FS treatments.

**Table 3:** Proportion of soil organic C and total N as biomass C (C_mic_:C_org_) and biomass N (N_mic_:N_tot_) and the metabolic quotient (*q*CO_2_) as affected by the treatments

Treatments	C_mic_:C_org_ (%)	N_mic_:N_tot_ (%)	*q*CO_2_ (μg CO_2_ mg C_mic_ g^−1^ soil h^−1^)
MF	0.80 b	1.16 ab	1.35 b
1SF	1.16 a	1.11 ab	1.17 b
2SF	1.04 ab	1.47 a	1.25 b
4SF	1.03 ab	1.33 ab	1.36 ab
8SF	0.99 ab	0.97 b	1.70 a

Means followed by the same letter(s) within the same column are statistically the same at *p* ≤ 0.05 (LSD-test)

MF, mineral fertilization recommended for the crop; 1FS, sewage sludge based on the nitrogen concentration that provides the same amount of N as in the mineral fertilization; 2FS, two; 4FS, four; 8FS, eight times the N recommended sewage sludge dosage

### Data analysis

**Fig. 2:**
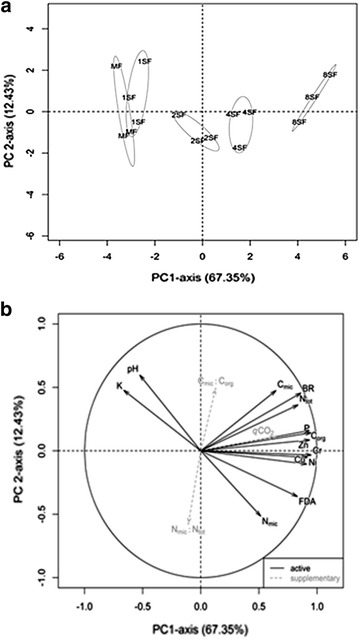
Scoring plot of treatments (**a**) and loading plot of variables (**b**) ordinated in PCA. The variance percentage explained by each component is given in parenthesis. Abbreviations of variables are explained in Materials and methods. MF, mineral fertilization; 1FS, dose 1 of sewage sludge; 2FS, dose 2 of sewage sludge; 4Fs, dose 4 of sewage sludge and 8FS, dose 8 of sewage sludge

The variables C_org_, BR, *q*CO_2_, Cr, Cu, P, Ni, Zn and FDA correlated in a significant and positive way with to each other. N_tot_ was positively correlated with C_mic_ and with all the variables above except for FDA and *q*CO_2_. C_mic_ was positively correlated with BR, C_mic_:C_org_, C_org_, Cr, Ni, N_tot_, P_av_, while N_mic_ was positively correlated with N_mic_:N_tot_, Cr, Ni and negatively correlated with K. K was also negatively correlated with N_tot_, Ni, Cr, Cu, FDA and positively correlated with pH. The PCA was used to visualize the response patterns of the microbiological and chemical parameters of the soil to different doses of sewage sludge and mineral fertilizer (Fig. 2a). This analysis showed that were retained four components (PC1, PC2, PC3 and PC4) that explained 67.35, 12.43, 8.9 and 4 % of the total variance, respectively. PC1 and PC2 were chosen to draw the score and loading plots since together they explained 79.78 % of the total variance together (Fig. 2a). The Cr, P, Zn, C_org_, Ni and Cu variables presented positive correlations with PC1 (r > 0.90). In comparison with PC1, PC2 was significantly (*p* < 0.05) weighted by pH and N_mic_ but with weak correlation. The scoring plot shows the position of the different treatments in the orthogonal space defined by the PC1 and PC2. They clearly discriminated the treatments 1FS, 2FS, 4FS and 8FS along this axis, but clustered 1FS and MF as one group (Fig. 2b).

## Discussion

### Soil chemistry

Although presenting some variations between the doses of sludge, the C_org_, N_tot_, P_av_ and K contents and those of some micronutrients did not vary between the control and the 1FS dose, calculated according to plant nitrogen requirement and the N content of the residue. The data for P_av_ found in the soil treated with 1FS were obtained after six sludge applications followed by two years with no application, suggesting that it is a highly residual component. However, it is not known if the P_av_ levels in the soil in that treatment could increase to environmentally dangerous levels over the years, even applying the dose considering as ideal. Thus considering the high values obtained for P_av_ in the soil with the higher sludge doses, it is important to constantly monitor this element in the soil. Other authors also observed an increase in the P content available in the soil after applying sewage sludge (Suhadolc et al. 2010; Ailincai et al. 2007; Gilmour et al. 2003).

All the values obtained for heavy metals were below the mandatory limits allowed by Brasil (2006). The pH of the soil in all the treatments and the greater quantities of organic matter, principally for the higher sludge doses, may have reduced the solubility of the heavy metals. The greatest amounts obtained for Cr in this study with the increasing sludge doses corroborated the results obtained by Ailincai et al. (2007) in soils amended with 40 and 60 Mg ha^−1^ of sewage sludge. Cr has not been proven to be essential in plant nutrition, but is required by microorganisms in some specific metabolic processes (Castilho et al. 2001). Increases in Ni concentrations in the soil due to the application of sewage sludge have also been shown by other authors (Ahumada et al. 2010; Oleszczuk 2008), but in general the concentrations found were much greater than those found in the present study. It has been suggested that nickel is an essential plant micronutrient, since it is part of the active site of some enzymes, such as, for example, urease (Eskew et al. 1984). The higher content of Zn found in the soils treated with MF as compared to those treated with 1FS, could be related to the large contents of this element in the fertilizers used. The Cu content in the soil treated with 8FS was 125 % greater than in the soil treated with 1FS. Elements such as copper and zinc are essential constituents of physiological processes in all living organisms, including microorganisms (Nies 2004). The micronutrients were all positively correlated with C_org_ showing the accumulation of metals in the soil organic matter (Table 4).

**Table 4:** Correlations coefficients between some microbial and chemical factors

	C_mic_	N_mic_	BR	FDA	*q*CO_2_	C_org_	C_mic_:C_org_	N_mic_:N_tot_	Cu	Cr	Ni	Zn	N_tot_	K	P
N_mic_	0.33														
BR	0.76**	0.19													
FDA	0.29	0.45	0.55*												
*q*CO_2_	0.1	0.04	0.71**	0.59*											
C_org_	0.54*	0.27	0.88***	0.77***	0.77***										
C_mic_:C_org_	0.82***	0.16	0.31	−0.19	−0.38	−0.03									
N_mic_:N_tot_	−0.06	0.79***	−0.36	−0.06	−0.4	−0.33	0.11								
Cu	0.39	0.51	0.7**	0.82***	0.67**	0.9***	−0.16	−0.06							
Cr	0.64*	0.57*	0.78***	0.76**	0.53*	0.82***	0.19	−0.04	0.87***						
Ni	0.64*	0.6*	0.77***	0.74**	0.52*	0.78***	0.22	0.03	0.77***	0.94***					
Zn	0.49	0.32	0.83***	0.8***	0.75**	0.97***	−0.08	−0.3	0.94***	0.86***	0.8***				
N_tot_	0.77***	0.28	0.89***	0.49	0.49	0.79***	0.37	−0.25	0.69**	0.77***	0.71**	0.75**			
K	−0.36	−0.61*	−0.35	−0.66**	−0.17	−0.4	−0.13	−0.22	−0.52*	−0.72**	−0.68**	−0.44	−0.52*		
P_av_	0.59*	0.31	0.89***	0.78***	0.72**	0.98***	0.04	−0.29	0.87***	0.84***	0.81***	0.96***	0.79***	−0.47	
pH	−0.01	−0.37	−0.23	−0.66**	−0.39	−0.5	0.35	−0.09	−0.49	−0.39	−0.4	−0.48	−0.25	0.53*	−0.49

BR, basic respiration; FDA, fluorescein diacetate activity; *q*CO_2_, metabolic quotient

* *p* < 0.05; ** *p* < 0.01; *** *p* < 0.001

### Microbial biomass and activity

Although several authors have reported that C_mic_ is very sensitive to residue input, in the present study a marked difference between treatments was only obtained when comparing treatments MF and 8SF, despite the increase in C_org_ content with increase in sludge dose. These results are in agreement with those obtained by Stark et al. (2008), where the authors verified that doubling the addition of organic matter did not result in a proportional increase in soil microbial biomass. Despite this, the values for C_mic_ correlated positively with the C_org_ and N_tot_ contents, as also observed by Böhme et al. (2005), which could indicate a relationship between the microbial biomass C and the labile C and N fractions in the soil, which were not evaluated. The C_mic_ also correlated positively with the Cr, Ni and P contents, showing that these elements could have been important for the soil microorganisms. The highest values for N_mic_, or the N immobilized by microorganisms, were obtained in the three largest sludge doses. Although some authors also obtained a positive correlation between N_mic_ and N_tot_, this did not occur in the present study. The absence of correlation between N_mic_ and N_tot_ could indicate that the microbial biomass N was associated with a larger quantity of readily mineralizable N_org_ in the larger sludge doses. The positive correlation between N_mic_ and the contents of the micronutrients Cr and Ni could also highlight the importance of these elements in increasing the immobilization of N by the microorganisms.

In general, the addition of organic materials enhances respiratory activity, because the organic residues are energetic substrates consumed during the oxidative metabolism of the heterotrophic soil microbiota (Bhattacharyya et al. 2001). The similarity in the C_org_ contents of the MF, 1FS and 2FS treatments could have contributed to the absence of a significant difference in BR between these treatments. Despite this, as can be seen in Table 4, there was a high positive correlation between BR and C_org_. This result could be related to the greater amounts of Cu, Cr, Ni, Zn, N_tot_ and P obtained with the increase in C_org_ content, since these elements have high positive correlations with BR.

The total microbial activity in terms of fluorescein diacetate has been used to determine the amount of active microflora producing extracellular enzymes (Adam and Duncan 2001), such as protease, lipase and esterase, all involved in the microbial decomposition of organic matter in the soil. For this reason, it has been considered a good indicator of the overall biological activity (Dick 1994). Hydrolysis was already found amongst a wide array of primary decomposers such as bacteria and fungi (Schnürer and Rosswall 1982). Practically all the chemical soil factors (Cu, Cr, Ni, Zn, C_org_ and P) controlling BR had significant effects on the soil FDA hydrolysis, which could have led to the positive correlation between these two parameters. This suggests that both the soil FDA hydrolysis and the BR were equally efficient in demonstrating the effects of sludge doses added to the soil on the microbial soil activity. However, the FDA hydrolysis did not show a positive correlation with C_mic_, as already observed by Perucci (1992). This lack of correlation could have occurred due to a change in composition of the microorganisms in the areas supplemented with the largest sludge doses. According to Marschner et al. (2003) changes in microbial community composition are often observed after addition of organic or inorganic amendments in the soil.

A high C_mic_:C_org_ ratio is indicative of labile C accumulation in the soil, which is a favorable environment for microbial growth and, in general, presents a positive correlation with the C_org_ of the soil, which did not occur in the present study. This absence of correlation could indicate equal availabilities of readily mineralizable C in all the treatments, despite the differences in the total contents. According to Anderson and Domsch (1986) larger ratios imply in increased availability of fresh substrates, while smaller ratios imply in reduced availability. The N_mic_:N_tot_ ratio usually reflects the active N pool, being related to soil N availability and mineralization. However, the absence of correlation between this index and all the variables measured, including C_org_ and N_tot_, could lead us to presume that other parameters, not evaluated in this study, were responsible for the greater N_mic_:N_tot_ ratios obtained with the higher sludge doses, as for example, the potential mineralization of soil organic nitrogen.

The *q*CO_2_ takes both BR and C_mic_ into consideration, and has been cited as a better indicator of microbial metabolic efficiency than BR alone (Hu et al. 2011). The higher *q*CO_2_ values obtained with the highest sludge dose could indicate that the microorganisms were forced to degrade stable organic matter to get new available substrate, even though this is an energetically expensive process. According to Tarrasón et al. (2010) the presence of less stable organic matter in sewage sludge could lead to an increase in the metabolic quotient. According to Anderson and Domsch (1990) the *q*CO_2_ should ideally show a strong negative correlation with the C_mic_:C_org_ ratio. However in the present study there was no correlation at all between these two indexes, a situation also encountered by other authors (Chander et al. 2001). Similarly there was no negative correlation between C_mic_, N_mic_ and the *q*CO_2_ index. Thus the high *q*CO_2_ values obtained in the present study with the highest sludge doses could, in fact, be related to the presence of a more active microbial biomass in the decomposition of organic compounds which were used as energy and carbon sources by the soil microbiota, and not indicate metabolic stress. Mattana et al. (2014) showed that the higher values found for *q*CO_2_ in soil amended with sludge-derived materials was probably more due to increased microbial growth than to a stress response, as also described by Renella et al. (2007). Furthermore, the positive correlation between *q*CO_2_ and the variables of BR and FDA hydrolysis could also indicate that the higher values obtained for *q*CO_2_ in the treatments 4FS and 8FS were more likely due to greater organic matter decomposition activity than to a stress response. The positive correlation between *q*CO_2_ and all the soil elements except K, demonstrated that changes in this index were not only controlled by the C concentration in the soil, as also observed by Spohn and Chodak (2015). Logically further studies are required to assess the *q*CO_2_ during corn cultivation, when more C becomes available to the microorganisms, due to exudates from the roots. Another factor to ponder on with respect to the *q*CO_2_ values is that considering the high degree of microbial functional redundancy the microbial population could have adapted to the higher concentrations and/or different types of organic substrates with respect to less specific processes, or there could have been changes in the structure or composition of the microflora, resulting in a predominance of low energy efficient microorganisms (Dilly and Munch 1998). However, according to Schimel and Schaeffer (2012), the influence of changes in the structure of a microbial community on the measureable differences in an ecosystem process is a question that is still not completely understood. Another factor to be considered are the variations between C_tot_, N_tot_ and P_av_ contents in the soil, which was extremely different for the different sludge doses. Despite the growing evidence that various elements can interact to affect the biomass and microbial activity, little is yet known about how interactions between C, N and P may influence the structure of the microbial community (Fanin et al. 2015). Some studies have demonstrated differentiated responses amongst different groups of microorganisms with respect to the elements C, N and P in the soil (Krashevska et al. 2010). With respect to the addition of sewage sludge to the soil, this type of study is still an open door for a variety of questions.

The PCA of the soil variables assessed discriminated the treatments with sewage sludge and clustered the 1FS and MF treatments. Most of the variables were ordinated with 8FS treatment. This demonstrates that although this sludge dose was well above that recommended for application in agricultural areas, it increased the microbial activity, as shown by the BR and FDA activity, which presented highly significant correlation with the chemical soil characteristics, with the exception of the pH and K content.

## Conclusion

The results of this study showed that over the years, the addition of crescent doses of an anaerobically digested household sewage sludge affected both the microbial activity and the chemical characteristics of the soil. In chemical terms, the greatest impact caused by the application of increasing sludge doses to the soil was the accentuated increase in the P_av_ and heavy metal contents, although these latter elements were below the mandatory limits allowed by Brazil (2006). However, there were no differences between the MF and 1FS treatments with respect to the heavy metal contents or the P_av_ of the soil, which fails to confirm the initial hypothesis that continued sludge applications, even when using doses considered adequate, could increase the P and heavy metal contents of the soil. In the same way there were no significant differences between the MF and 1FS treatments with respect to the microbiological parameters measured. Considering that the results for C_mic_ were the same for all treatments despite the differences between some of the parameters that measured the activity of the microorganisms, became apparent the need to study the structure and composition of the soil microflora in order to better understand the effect of soil supplementation with sewage sludge. This study was carried out with the cultivation of corn in a specific type of soil under tropical conditions. In order to better understand the dynamics of this type of sludge after its addition to the soil, it should be studied in different types of soil for longer periods of time and also under different types of soil management, as for example, the inclusion of a winter crop.
